# Lecanemab, Aducanumab, and Gantenerumab — Binding Profiles to Different Forms of Amyloid-Beta Might Explain Efficacy and Side Effects in Clinical Trials for Alzheimer’s Disease

**DOI:** 10.1007/s13311-022-01308-6

**Published:** 2022-10-17

**Authors:** Linda Söderberg, Malin Johannesson, Patrik Nygren, Hanna Laudon, Fredrik Eriksson, Gunilla Osswald, Christer Möller, Lars Lannfelt

**Affiliations:** 1 BioArctic AB, Warfvinges väg 35, 112 51 Stockholm, Sweden; 2 Dept. of Public Health/Geriatrics, Uppsala University, 751 85 Uppsala, Sweden

**Keywords:** Amyloid-beta species, Therapeutic antibodies, Lecanemab, Aducanumab, Gantenerumab

## Abstract

Immunotherapy against amyloid-beta (Aβ) is a promising option for the treatment of Alzheimer’s disease (AD). Aβ exists as various species, including monomers, oligomers, protofibrils, and insoluble fibrils in plaques. Oligomers and protofibrils have been shown to be toxic, and removal of these aggregates might represent an effective treatment for AD. We have characterized the binding properties of lecanemab, aducanumab, and gantenerumab to different Aβ species with inhibition ELISA, immunodepletion, and surface plasmon resonance. All three antibodies bound monomers with low affinity. However, lecanemab and aducanumab had very weak binding to monomers, and gantenerumab somewhat stronger binding. Lecanemab was distinctive as it had tenfold stronger binding to protofibrils compared to fibrils. Aducanumab and gantenerumab preferred binding to fibrils over protofibrils. Our results show different binding profiles of lecanemab, aducanumab, and gantenerumab that may explain clinical results observed for these antibodies regarding both efficacy and side effects.

**Supplementary Information:**

The online version contains supplementary material available at 10.1007/s13311-022-01308-6.

## Introduction

According to the amyloid hypothesis, amyloid-beta (Aβ), the main constituent of extracellular plaques found in Alzheimer’s disease (AD) brains [1, 2], initiates the disease process and is therefore an attractive target for therapeutic intervention [3].

Aβ exists as various species, including monomers, soluble aggregates of varying size (e.g., oligomers and protofibrils), and insoluble fibrils in plaques [4, 5]. Studies of Aβ with the Arctic mutation showed that the peptide had an increased propensity to form soluble Aβ protofibrils and data indicated that these protofibrils were neurotoxic and contributed to the disease process [6–8]. Furthermore, it has been shown that soluble Aβ aggregates are more neurotoxic than monomers and insoluble fibrils [9, 10]. It could therefore be hypothesized that removal of these soluble Aβ aggregates would represent an effective approach for the treatment of AD [11, 12].

Immunotherapy has emerged as a promising treatment option for AD, although many challenges remain. Several monoclonal antibodies have entered clinical trials with varying degree of success. The clinical program of bapineuzumab [13], a monoclonal antibody with a high affinity for all forms of Aβ, was terminated since the desired clinical effect was not achieved [14]. Solanezumab was developed to target the mid-region of soluble, monomeric Aβ. In phase 3 studies, solanezumab failed to meet primary clinical endpoints [15]. Crenezumab, a monoclonal IgG4 antibody, which binds both monomeric and oligomeric forms of Aβ, was investigated in a phase 3 trial with similar results [16, 17]. Results from the clinical trials with solanezumab and crenezumab showed no, or limited, clinical effect signals and limited plaque clearance.

There are currently four monoclonal antibodies targeting Aβ in late-phase clinical development, lecanemab, aducanumab, gantenerumab, and donanemab. All four are monoclonal IgG1 antibodies targeting aggregated forms of Aβ. Lecanemab is a humanized version of the murine mAb158 antibody targeting soluble Aβ aggregates (oligomers and protofibrils) with high selectivity [10, 18, 19]. mAb158 was generated at Uppsala University, and lecanemab was further developed by BioArctic and Eisai. Lecanemab showed profound plaque clearance and efficacy signals in a phase 2b clinical study [20]. Aducanumab, developed by Biogen, is a recombinant human antibody that binds to amino acids 3–7 of the Aβ peptide. Development of aducanumab was halted in 2019 after analysis of the data from two phase 3 trials indicated that the primary endpoint would not be met. Further analysis of the data showed profound plaque clearance and an efficacy signal supporting progression of the clinical program, and aducanumab was approved in the USA by FDA in 2021 [21–24]. Gantenerumab was identified from a human combinatorial antibody library and is being developed by Roche. It differs from the other three in that it targets both the N-terminal, 3–11, and mid-regions, 18–27, of the Aβ peptide [25]. Two phase 3 clinical trials with gantenerumab were stopped in 2014 after an interim futility analysis reported no efficacy on primary or secondary endpoints. The antibody has since then reentered clinical development and is currently in two phase 3 trials with readouts expected in the fall of 2022.

Data from late phase clinical trials indicates that Aβ immunotherapy can have positive effects. However, amyloid-related imaging abnormalities, mainly with edema (ARIA-E), have been observed. The main risk factors identified for developing ARIA-E have been antibody dose and the presence of the apolipoprotein E4 (ApoE4) allele [26]. The exact mechanism for the occurrence of ARIA-E has not been elucidated, but probable explanations involve direct binding of Aβ antibodies to cerebral amyloid angiopathy (CAA) [27, 28], or accelerated formation of CAA [29]. CAA is a pathology consisting of fibrillar Aβ, mainly Aβ1-40 [30], deposited in the blood vessel walls and is a common occurrence in AD [31, 32]. ARIA-E has been observed with the following frequencies: lecanemab 10% [20, 33], aducanumab 35% [21–24], gantenerumab 30% [23], and donanemab 27% [34].

In the present study, we report on in vitro, side-by-side comparisons of binding characteristics of three of the antibodies in late-phase clinical development, lecanemab, aducanumab, and gantenerumab. Donanemab was excluded since it does not bind N-terminal full-length Aβ, the peptide used in our studies [34, 35]. Binding of the antibodies to Aβ monomers, cross-linked oligomers, small and large protofibrils, and fibrils (Table 1) have been examined using inhibition ELISA, immunodepletion, and surface plasmon resonance (SPR). The data, while corroborating that all three antibodies are highly aggregate selective, show also that there are distinct differences. Aducanumab and gantenerumab demonstrated selectivity towards fibrils over protofibrils, whereas lecanemab showed stronger binding to protofibrils than to fibrils. These data are in line with previous published data on mAb158, the murine precursor of lecanemab [18]. To our knowledge, this is the first study where the binding profiles of the three antibodies have been compared side-by-side. It is possible that the differences seen in the binding profiles of lecanemab, aducanumab, and gantenerumab may explain the variation in efficacy and frequency of ARIA-E observed in the clinical trials.

**Table 1:** Size comparison of Aβ species used in this study

Aβ species	Approx. size (kDa)
**Monomer**	
Aβ1-28	3.3
Aβ1-40	4.3
**Oligomer**	
2–3-mer	9–14
6–8-mer	27–36
8–12-mer	36–54
**Protofibril**	
Small	75–400
Large	300–5000
**Fibril**	Insoluble

## Material and Methods

### Generation of Anti-Aβ Antibodies

Based on publicly available sequence information on aducanumab [36] and gantenerumab [37], analogues were recombinantly produced. Gantenerumab was produced transiently in HEK293 cells using the Absolute Antibody HEXpress™ antibody expression platform and proprietary vectors (Absolute Antibody, Oxford, UK). Purification was done by affinity chromatography and size exclusion chromatography (SEC). The final buffer was PBS, pH 7.2 (Gibco, Cat. No. 20012–019). Aducanumab was produced transiently in CHOK1SV GS-KO cells using the single gene GS expression vectors pXC-184 and pXC-17.4 (Lonza Biologics, Cambridge, UK). Purification was done by protein A affinity chromatography and SEC and the final buffer was PBS, pH 7.2. The purity was estimated to be above 98% by SEC and by SDS-PAGE under denaturing conditions. Lecanemab was provided by Eisai Co., Ltd.

### Preparation of Different Aβ Species

Aβ peptides were purchased from Bachem (Bachem, Inc., Switzerland). Lyophilized peptides of Aβ1-40, Aβ1-42, and Aβ1-28 were reconstituted in 10 mM NaOH, 0.005% Tween-20, pH 11 to a concentration of 100 µM and stored at − 80 °C until used. Protofibrils were prepared by diluting Aβ1-42 peptide stock solution twofold with 2 × PBS (100 mM phosphate, 300 mM NaCl, pH 7.4) and incubating the mixture at 37 °C for 45 min. Protofibrils were purified from fibrils by centrifugation at 16,000 × g for 10 min and further purified from monomers by SEC on a Superdex 75 increase 3.2/300 column (Cytiva, Uppsala, Sweden) using a mobile-phase composed of 50 mM phosphate, 150 mM NaCl, 0.1% tween-20, pH 7.4 with a flow-rate of 0.08 ml/min. Protofibrils were defined as Aβ1-42 aggregates that remains in the supernatant after centrifugation at 16,000 × g and elutes in the void volume of a Superdex 75 column. The size ranges of purified protofibrils were determined by SEC using a Superdex 200 increase 3.2/300 column (Cytiva, Uppsala, Sweden) and a Superose 6 increase 3.2/300 column (Cytiva, Uppsala, Sweden) using the same conditions as described above. Large protofibrils were defined as Aβ species that eluted, according to globular molecular standards, between 300 and 5000 kDa and small protofibrils eluted with a size of 75–200 kDa (Supporting information 1). Fibrils were prepared by diluting Aβ1-42 peptide solution stock twofold with 2 × PBS and incubating the mixture at 37 °C for 48 h. An aliquot was removed, centrifuged (16,000 × g), and the supernatant analyzed by SEC to determine that the fibril formation had gone to completion. Aβ1-42 oligomers were stabilized covalently using a modified photo-induced cross-linking of unmodified proteins (PICUP) protocol [38]. Briefly, Aβ1-42 stock solution was diluted twofold with 2 × PBS and incubated for 5–15 min at 37 °C before Tris(2,2-bipyridyl)dichlororuthenium(II) hexahydrate (RuBpy) (Sigma-Aldrich, Saint Louis, USA) and ammonium persulfate were added to a final concentration of 0.25 mM and 1%, respectively. The mixture was irradiated for 5 s whereafter the reaction was quenched by removal of the peptide from the reaction mixture using a Zeba spin desalting column 7 k MWCO (Thermofisher, Waltham, USA). The oligomers were purified on a Superdex 75 increase column and fractions collected. Size and concentration were estimated by SEC using globular protein standards and a calibration curve of an Aβ protofibril standard with known concentration.

### Inhibition ELISA

**Fig. 1:**
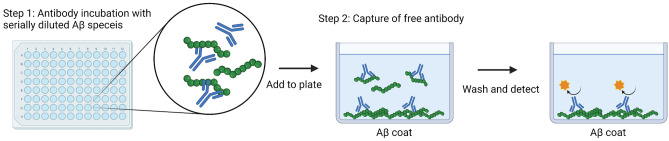
Principle of the inhibition ELISA. Two steps are involved in the inhibition ELISA. Step one: incubation of Aβ species with the investigated antibodies for specific binding. Step two: the antibody-antigen mixtures from step one, containing complexes and free antibodies, are added to the plate coated with Aβ-protofibrils to allow binding of free antibody. Antigen binding in step one will then consequently reduce binding to the coated Aβ-protofibrils. As a result of prior binding of sample antigen to primary antibody, the reaction in the ELISA plate wells is reduced and the antigen concentration required to inhibit half of the maximum signal in the inhibition ELISA is defined as IC_50_, which was used as an estimate of the antibody’s affinity and selectivity for the investigated antigen

The inhibition ELISA was performed as previously described [39]. Antibodies (50 ng/ml) were preincubated for 45 min at 900 rpm with either threefold serially diluted Aβ monomers or protofibrils with starting concentrations of 25,000 nM and 250 nM, respectively. For the oligomers, 6.3 ng/ml antibody and threefold serially diluted oligomer fractions, 2–3-mer, 6–8-mer, and 8–12-mer, with starting concentration of 179, 368, and 437 nM, respectively, were preincubated for 45 min at 900 rpm. The preincubated antibody/Aβ mixture was added to an ELISA plate coated with 11 µg/ml Aβ protofibrils and incubated for 25 min without shaking. The ELISA plate was washed, and captured antibody was detected with an ALP-conjugated anti-human IgG (Mabtech, Sweden). Absorbance values (OD_405_) were plotted against log10 of Aβ concentrations, and the values were normalized against the absorbance value obtained for the lowest Aβ concentration used. The principle of the inhibition ELISA is illustrated in Fig. 1.

### Immunodepletion of Synthetic Aβ Protofibrils

Each antibody was tenfold serially diluted in five steps with a starting concentration of 1000 ng/ml and incubated with 10 pM protofibrils for 2 h at 22 °C in a thermomixer at 1100 rpm. After 2 h incubation, magnetic protein A beads (Invitrogen, Waltham, USA) were added to the reaction, and the mixture was incubated for an additional 30 min before the beads were separated from the samples with a magnet, and the Aβ protofibril content in the supernatant was analyzed by MSD using a Aβ protofibril selective assay. Briefly, samples were added to an MSD plate coated with 0.5 μg/ml mAb158 antibody. The plate was washed, incubated with 0.5 µg/ml biotinylated anti-Aβ antibody mAb1C3 [19, 39], washed again, and incubated with streptavidin SULFO-TAG. Measurement was preformed using an MSD SECTOR instrument (MSD, Rockville, USA). Due to the minor differences in the molecular weight of the antibodies, the highest final antibody concentration for lecanemab, aducanumab, and gantenerumab was 6800, 6850, and 6830 pM, respectively.

### Preparation of Brain Extracts from Alzheimer’s Disease Brain

Brain samples were obtained from the Netherlands Brain Bank (NBB) (Netherlands Institute for Neuroscience, Amsterdam (Open access: www.brainbank.nl). All material was collected from donors from whom written informed consents for brain autopsy were provided. The informed consent form of the NBB meets all current legal and ethical requirements for brain autopsy, tissue storage, and use of tissue and clinical data for scientific research worldwide. The Swedish Ethical Review Authority (no. 2020–00,527) approved the study. Fresh frozen temporal cortex tissue, from three individuals with confirmed diagnosis of AD, was extracted in TBS buffer supplemented with protease and phosphatase inhibitors (Roche) in a 1:10 weight/volume (w/v) ratio using a Potter–Elvehjem homogenizer. The extracts were then centrifuged at 16,000 × g for 60 min at + 4 °C, and the supernatants collected.

### Immunodepletion of Soluble Protofibrils from AD Brain Extracts

Immunodepletion was performed by mixing serially diluted antibodies (lecanemab, aducanumab, gantenerumab), final concentration 10,000, 1000, 100, 10, 1, 0.1, and 0.01 ng/ml, with brain extracts, final protofibril concentration 25–35 pM, in 1% Blocker A (Meso Scale). Protofibril concentration was determined using the protofibril specific assay described above. The samples were incubated for 2 h at 22 °C in a thermomixer at 1100 rpm, whereafter magnetic protein A beads (450 µg/reaction) were added to the reaction and the samples were incubated for an additional 30 min. The antibody/Aβ complex was immunoprecipitated with the bead when placed in a magnetic holder, the supernatant was collected, and the protofibril content analyzed using the mAb158/mAb1C3 protofibril assay described above. Due to the minor differences in the molecular weight of the antibodies, the highest final antibody concentration for lecanemab, aducanumab, and gantenerumab was 68,000, 68,500, and 68,300 pM, respectively. Samples with protofibril concentrations below LLOQ were set to 0% and the data normalized against the lowest antibody concentration in the dilution series before the data was fitted to a nonlinear regression with sigmoidal dose response (variable slope) curve. EC_50_ values were calculated by GraphPad Prism (when applicable).

### Affinity Measurements by Surface Plasmon Resonance

Binding kinetics and affinity measurements were performed with a Biacore 8 K or 8 K + instrument (Cytiva, Uppsala, Sweden). Monomer binding was measured using single cycle kinetics (SCK) and SCK using capture. For SCK, 5 µg antibody diluted in 10 mM acetate buffer, pH 4.5, was immobilized on a CM5 chip using general coupling chemistry with the surface preparation method “Immobilization low levels.” For SCK using capture, a human antibody capture chip was prepared according to manufacturer’s instructions using the Cytiva human antibody capture kit. Aβ1-28 monomer, two- or fourfold diluted, in 5 steps, from 10,000 nM, was then injected over the antibodies. Dissociation time was set to 600 s. Two experiments were performed for each setup, with four SCK injections per antibody in each experiment. An injection of 3 M MgCl_2_ was used to regenerate the surface between each cycle. The binding curves were fitted to a 1:1 interaction model. For protofibril and fibril binding, Aβ species were immobilized directly on a CM5 chip using general coupling chemistry with the surface preparation method “Immobilization low levels.” Protofibrils and fibrils were immobilized in 10 mM acetate buffer, pH 4.5 and pH 4.0, respectively. Antibodies were injected over the Aβ species diluted twofold in 5 steps, and the highest concentrations used were 1, 10, or 100 nM. Dissociation time was set to 1800s for Aβ1-42 protofibrils and 1200 s for Aβ1-42 fibrils. An injection of 3 M MgCl_2_ or 10 mM Glycine–HCl pH 1.7 was used to regenerate the surface between each cycle. The binding data was fitted to a bivalent analyte model.

## Results

### Binding to Aβ Monomers, Oligomers and Protofibrils Using Inhibition ELISA

Binding of lecanemab, aducanumab, and gantenerumab to different in vitro generated soluble species of Aβ, monomers, oligomers, and protofibrils, was investigated by inhibition ELISA.

**Fig. 2:**
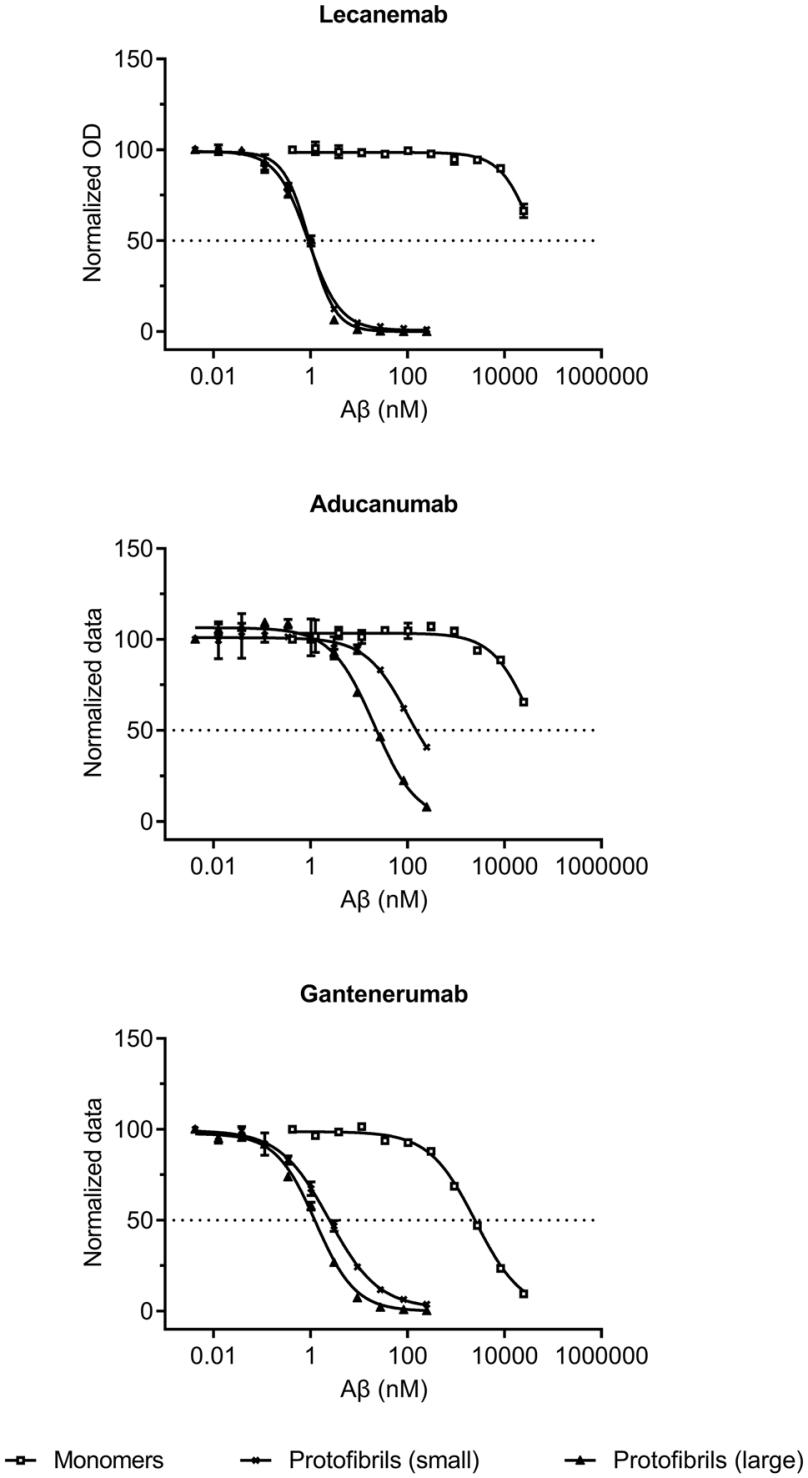
Results from inhibition ELISA with binding to monomeric Aβ and small and large protofibrils. Normalized and blank subtracted OD_405_ values were plotted against Aβ concentration. Curves represent mean ± SD for monomeric Aβ, squares, small protofibrils, black crosses, and large protofibrils, black triangles

**Fig. 3:**
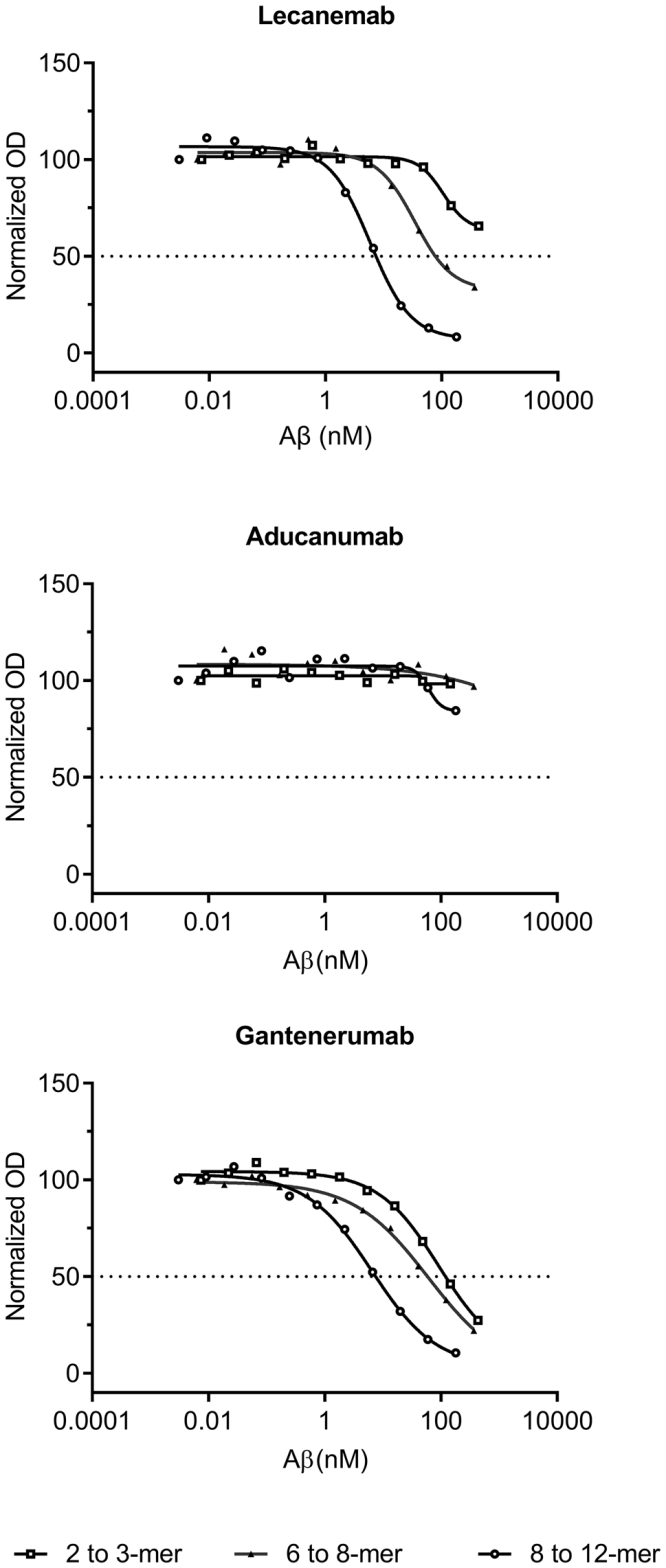
Results from the inhibition ELISA with small Aβ oligomers. Normalized and blank subtracted OD_405_ values were plotted against Aβ concentrations. Curves representing 8–12-mer, open circles, 6–8-mer, black triangles, and 2–3-mer, open squares

For all antibodies, IC_50_ values were in the µM range for monomeric Aβ (Fig. 2, Table 2). However, lecanemab and aducanumab both had IC_50_ values above > 25 µM, which indicated a very weak binding to monomers. Gantenerumab had an IC_50_ of 2.6 µM for Aβ monomers, suggesting a stronger binding to monomers. The lowest IC_50_ values for binding to both small and large protofibrils were obtained with lecanemab with an IC_50_ of 0.8 nM. Aducanumab displayed a weak binding, relative to lecanemab and gantenerumab, to both the small and the large protofibrils, with an IC_50_ > 80 nM and 22 nM, respectively. Gantenerumab showed a stronger binding to the large protofibrils with an IC_50_ of 1.3 nM as compared to the smaller protofibrils with an IC_50_ of 2.5 nM. The differences in the binding strengths between the antibodies were most evident when small protofibrils were investigated to which lecanemab demonstrated a threefold stronger binding than gantenerumab and > 100 times stronger binding than aducanumab. Lecanemab and gantenerumab presented similar binding to oligomers, but the binding strength was lower compared to binding to protofibrils and decreased with reduced oligomer size. For the smallest oligomers, such as dimers and trimers, gantenerumab showed an approximately tenfold stronger binding than lecanemab (Fig. 3, Table 3). Aducanumab did not bind to the oligomers at the concentration range used.

**Table 2:** Binding to Aβ monomers and small and large Aβ protofibrils by inhibition ELISA presented as mean ± SD

Antibody	Monomers IC_50_ (nM)	Small protofibril IC_50_ (nM)	Large protofibril IC_50_ (nM)
Lecanemab	> 25,000	0.80 ± 0.10	0.79 ± 0.20
Aducanumab	> 25,000	> 83	22.0 ± 2.0
Gantenerumab	2600 ± 130	2.5 ± 0.10	1.3 ± 0.10

**Table 3:** Binding to small Aβ oligomers by inhibition ELISA presented as mean ± SD

Antibody	8 to 12-mer IC_50_ (nM)	6 to 8-mer IC_50_ (nM)	2 to 3-mer IC_50_ (nM)
Lecanemab	6.1 ± 0.20	> 41	> 440
Aducanumab	> 180	> 370	> 440
Gantenerumab	5.7 ± 1.1	> 41	> 49

### Immunodepletion of Synthetic Aβ Protofibrils and Protofibrils from AD Brain Extracts

**Fig. 4:**
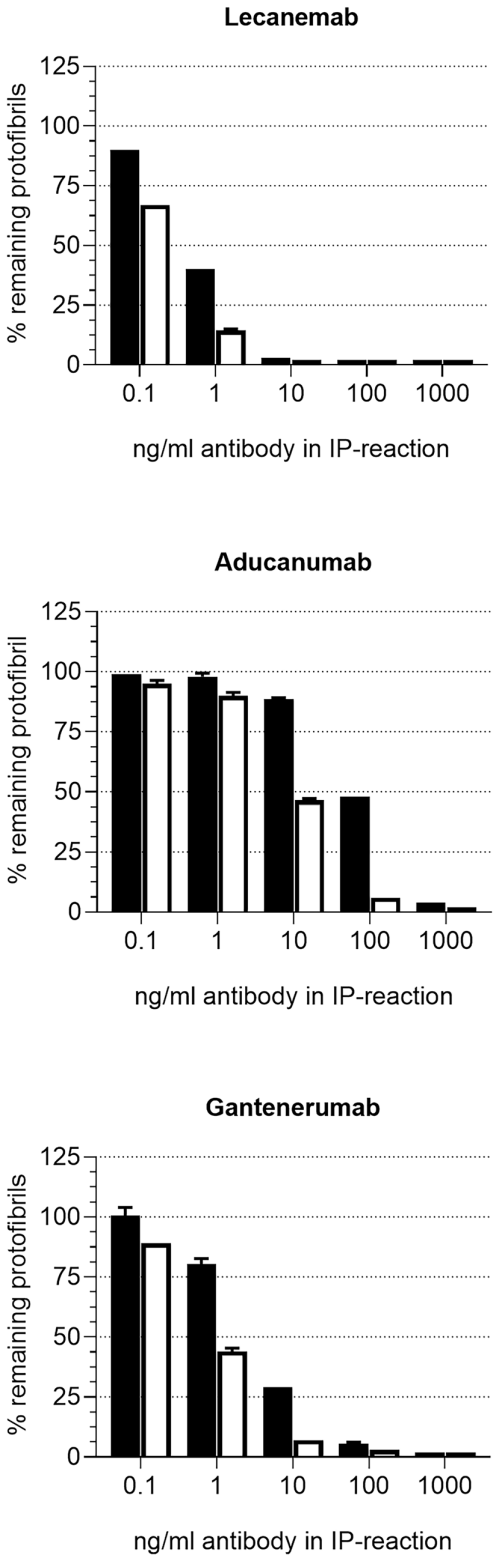
Evaluation of antibody binding to synthetic Aβ protofibrils in solution using immunodepletion. Antibodies, 0.67, 6.7, 67, 670, 6670 pM, were incubated with 10 pM of small (black columns) or large (white columns) Aβ protofibrils, followed by pull-down using magnetic protein A beads. Unbound Aβ protofibrils remained in the supernatant. Data expressed as % remaining protofibrils compared to bead control, and are presented as mean ± SD

Immunodepletion was employed to further evaluate how the antibodies bound protofibrils in solution. Serially diluted antibodies were allowed to interact with synthetic Aβ protofibrils and the antibody/Aβ complex was depleted with magnetic protein A beads. For lecanemab, a near complete depletion of the protofibrils (10 pM) was observed for antibody concentrations of 10 ng/ml and higher, with partial depletion observed at 1 and 0.1 ng/ml (Fig. 4). The EC_50_ of the depletion of protofibrils with lecanemab indicated a small preference for the large protofibrils over the small (Table 4). The concentration of aducanumab needed to achieve a near complete depletion of the protofibrils was 1000 ng/ml, with an EC_50_ of 630 and 61 nM for the large and small, respectively. Near complete depletion of the large protofibrils was achieved with 10 ng/ml gantenerumab, whereas 100 ng/ml was needed to deplete the small. Calculation of the EC_50_ for the depletion of the protofibrils showed that gantenerumab was ~ 5 times more effective in depleting the large protofibrils than the small.

**Table 4:** Immunodepletion of synthetic Aβ protofibrils

	Lecanemab *EC*_*50*_* (pM)*	Aducanumab *EC*_*50*_* (pM)*	Gantenerumab *EC*_*50*_* (pM)*
Small protofibril	5.3	631	27
Large protofibril	3.5	61	5.5

**Fig. 5:**
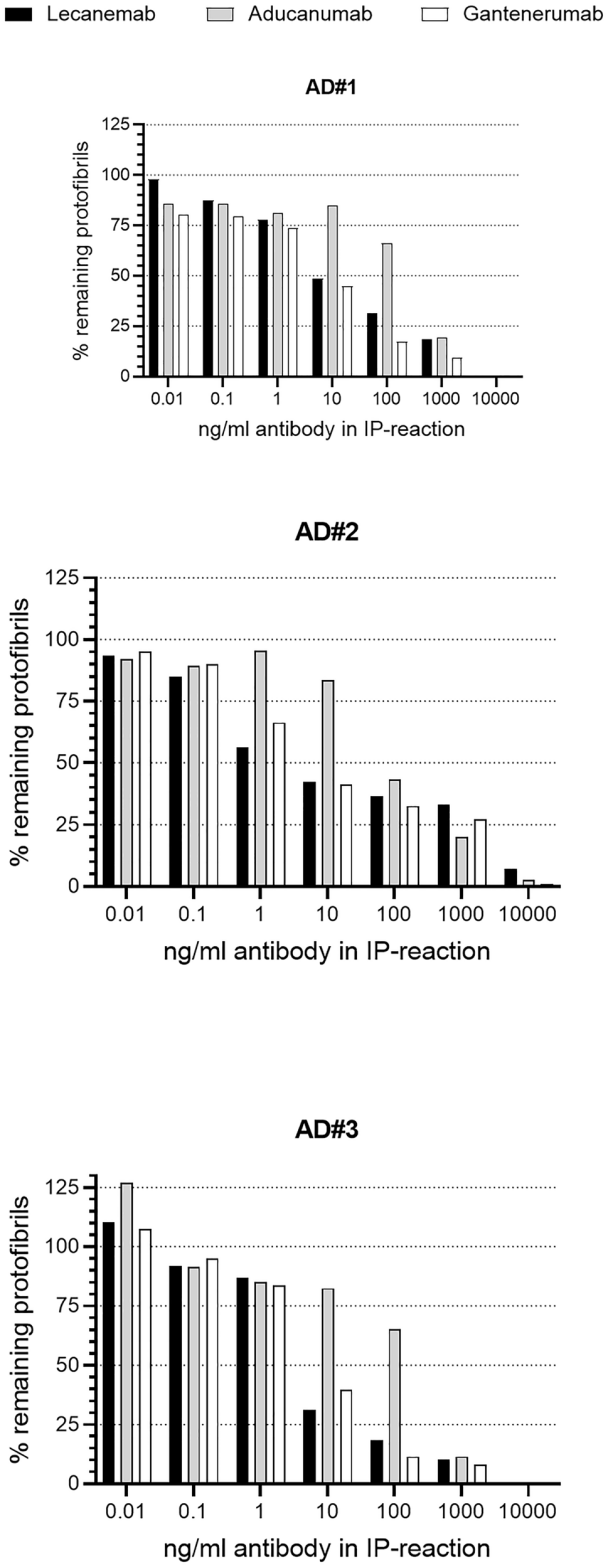
Immunodepletion of protofibrils from AD soluble brain extracts using lecanemab, aducanumab, and gantenerumab. Protofibrils remaining in the supernatant were analyzed using a protofibril specific assay (mAb158-1C3-bio). Data expressed as % remaining protofibril compared to bead control

Immunodepletion was also employed to investigate the binding of lecanemab, aducanumab, and gantenerumab to Aβ species in brain extracts from three AD donors with APOE E4/4 genotype. Titrating amounts of antibody was incubated with a fixed concentration of brain extract, the immunocomplex was depleted, and the percentage of protofibrils remaining in the supernatant was calculated, compared to bead control. The amount protofibrils in the supernatant was plotted against the antibody concentration (Fig. 5). The efficiency with which lecanemab and gantenerumab immunodepleted protofibrils were similar for each of the three donors, and reduced levels of protofibrils could be observed at 10 ng/ml antibody in the extracts from donors 1 and 3, and at 1 ng/ml for donor 2. In contrast, the concentration of aducanumab needed to reach an immunodepletion efficiency comparable to lecanemab and gantenerumab was 100 to 1000 ng/ml. EC_50_ values were calculated using the protofibril depletion data indicated that the EC_50_ for aducanumab was 12- to 26-fold higher compared to lecanemab and 9- to 30-fold higher compared to gantenerumab (Table 5).

**Table 5:** Immunodepletion of soluble Aβ protofibrils from AD brain extracts

AD case	Lecanemab *EC*_*50*_* (pM)*	Aducanumab *EC*_*50*_* (pM)*	Gantenerumab *EC*_*50*_* (pM)*
#1	96	1600	52
#2	46	570	61
#3	33	870	41

### Binding to Aβ Monomers, Protofibrils and Fibrils, Investigated by SPR

**Fig. 6:**
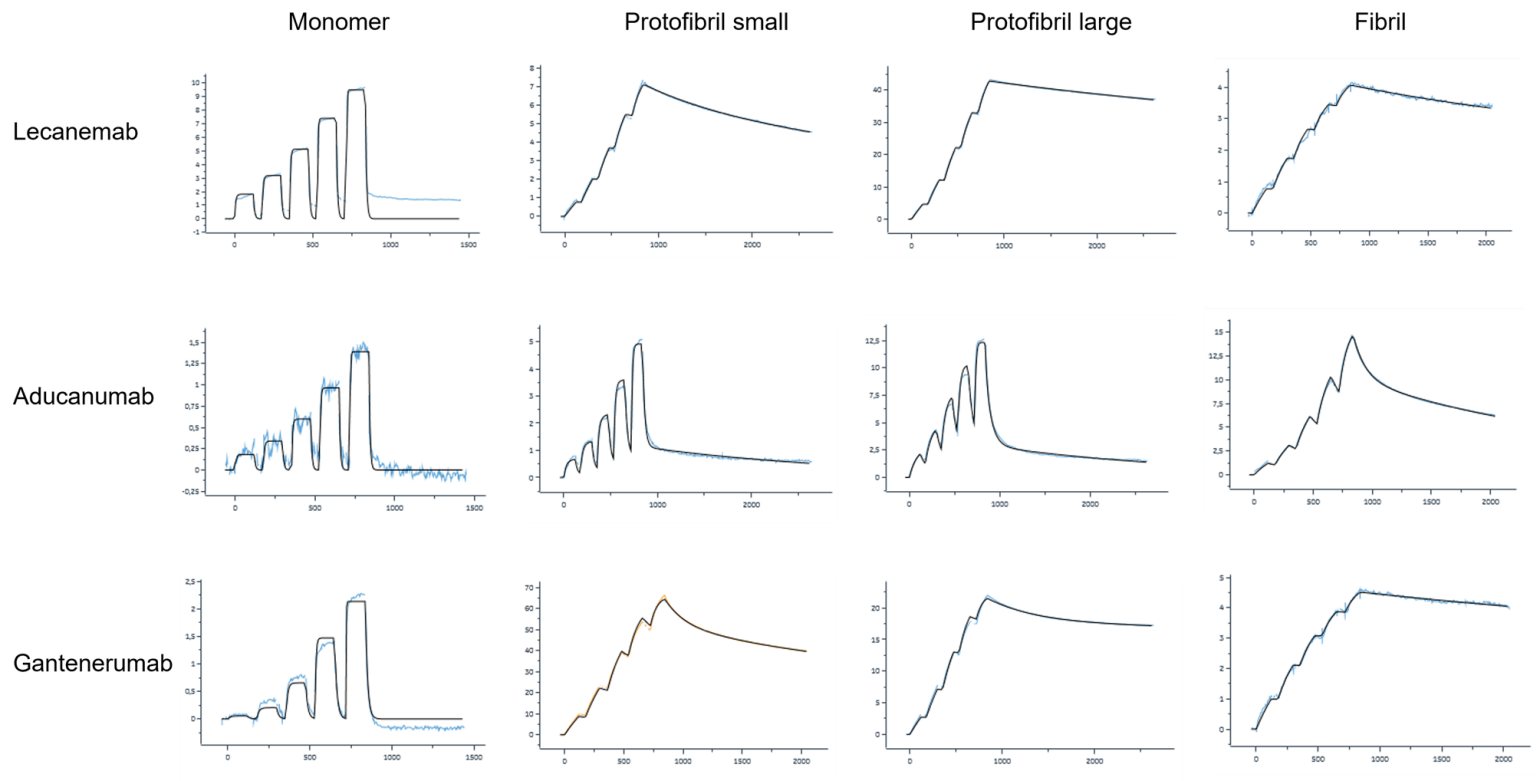
Representative SPR sensorgrams (blue curves) and curve fittings (black curves) of binding to Aβ monomers, small and large protofibrils, and fibrils for lecanemab, aducanumab, and gantenerumab

The kinetic properties of the binding of lecanemab, aducanumab, and gantenerumab to different Aβ species were investigated using surface plasmon resonance (SPR). Injection of Aβ monomers over immobilized antibody showed that all three bound the monomer with low affinity, lecanemab K_D_ of 2300 ± 910 nM, aducanumab 7300 ± 990 nM, and gantenerumab 1300 ± 480 nM. The association rate for the monomer binding to the antibodies was in the same order of magnitude, with a slightly faster association rate for lecanemab, 8.1 ± 6.9 × 10^4^, than for aducanumab and gantenerumab, 2.0 ± 0.36 × 10^4^ and 3.7 ± 1.3 × 10^4^, respectively. The low affinity of the antibodies for the monomer was driven by the dissociation rate. The dissociation rates for lecanemab and aducanumab were very fast, 1.6 ± 1.0 × 10^−1^ and 1.5 ± 0.11 × 10^−1^, respectively, whereas it was approximately 3 times slower for gantenerumab. Representative sensorgrams of the antibodies binding to monomers are shown in Fig. 6, and kinetic data are listed in Table 6.

**Table 6:** Antibody binding kinetics to Aβ monomers, protofibrils, and fibrils

**Aβ monomer**			
	***k***_**a**_ **(M**^**−1**^ **s**^**−1**^**)**	***k***_**d**_ **(s**^**−1**^**)**	***K***_**D**_ **(nM)**
Lecanemab	8.1 ± 6.9 × 10^4^	1.6 ± 1.0 × 10^−1^	2300 ± 910
Aducanumab	2.0 ± 0.36 × 10^4^	1.5 ± 0.11 × 10^−1^	7300 ± 990
Gantenerumab	3.7 ± 1.3 × 10^4^	4.8 ± 2.7 × 10^−2^	1300 ± 480

**Small Aβ protofibrilSmall Aβ protofibril**			
	***k***_**a1**_ **(M**^**−1**^ **s**^**−1**^**)**	***k***_**d1**_ **(s**^**−1**^**)**	***K***_**D1**_ **(nM)**
Lecanemab	5.3 ± 1.1 × 10^5^	4.5 ± 1.7 × 10^−4^	0.97 ± 0.66
Aducanumab	2.5 ± 0.53 × 10^7^	5.2 ± 1.7 × 10^−2^	2.2 ± 1.0
Gantenerumab	4.6 ± 1.1 × 10^5^	2.6 ± 1.0 × 10^−3^	5.7 ± 1.9

** Large Aβ protofibril**			
	***k***_**a1**_ **(M**^**−1**^ **s**^**−1**^**)**	***k***_**d1**_ **(s**^**−1**^**)**	***K***_**D1**_ **(nM)**
Lecanemab	7.6 ± 2.1 × 10^5^	1.1 ± 0.36 × 10^−4^	0.16 ± 0.07
Aducanumab	3.8 ± 0.56 × 10^7^	3.0 ± 0.56 × 10^−2^	0.79 ± 0.10
Gantenerumab	4.0 ± 0.91 × 10^5^	9.4 ± 2.7 × 10^−4^	2.5 ± 0.99

**Aβ fibril**			
	***k***_**a1**_ **(M**^**−1**^ **s**^**−1**^**)**	***k***_**d1**_ **(s**^**−1**^**)**	***K***_**D1**_ **(nM)**
Lecanemab	1.5 ± 0.47 × 10^5^	2.5 ± 0.91 × 10^−4^	1.8 ± 0.93
Aducanumab	2.1 ± 1.3 × 10^6^	6.2 ± 3.9 × 10^−3^	3.3 ± 2.2
Gantenerumab	1.4 ± 0.21 × 10^5^	9.4 ± 2.2 × 10^−5^	0.69 ± 0.16

Data presented as mean ± SD, *k*_*a1*_ apparent association rate constant, *k*_*d1*_ apparent dissociation rate constant, *K*_*D1*_ apparent dissociation constant

To investigate binding to Aβ protofibrils by SPR, the protofibrils were immobilized on a chip and the antibodies were subsequently injected over the surface. The data was fitted to a bivalent analyte model and the affinity (dissociation constant) reported as K_D1_. The simultaneous binding with both arms of the antibody results in a stronger interaction with the target, a phenomenon known as avidity. K_D1_ is the calculated apparent affinity of the initial binding with one arm.

All three antibodies bound to the protofibrils with high apparent affinity. The apparent affinity of lecanemab binding small and large protofibrils was determined to be K_D1_ of 0.97 ± 0.66 and 0.16 ± 0.07 nM, respectively. The increased affinity was driven mainly by the rate of dissociation which was approximately three orders of magnitude slower than for the binding to the monomer, 4.5 ± 1.7 × 10^−4^ s^−1^ and 1.1 ± 0.36 × 10^−4^ s^−1^, for the small and large protofibrils, respectively. The binding of aducanumab to the protofibrils was, unlike lecanemab, driven by a very fast apparent association rate, ka1 of 2.5 ± 0.53 × 10^7^ M^−1^ s^−1^ for small and 3.8 ± 0.56 × 10^7^ s^−1^ for large protofibrils. Aducanumab’s apparent dissociation rate when bound to the protofibrils was 5.2 ± 1.7 × 10^−2^ s^−1^ and 3.0 ± 0.56 × 10^−2^ s^−1^, for the small and large protofibrils, respectively, which was comparable to the dissociation rate of the monomer binding to gantenerumab. The fast apparent association and apparent dissociation rates associated with the binding of aducanumab to the protofibrils indicate that there is a rapid exchange of bound and unbound antibody, despite the high apparent affinity (2.2 ± 1.0 nM for small and 0.79 ± 0.10 nM for large protofibrils).

The apparent affinity of gantenerumab to the protofibrils was slightly weaker, 5.7 ± 1.9 nM for small and 2.5 ± 0.99 nM for large, than for lecanemab and aducanumab. Irrespective of protofibril size, the apparent association rate of gantenerumab was approximately one order of magnitude faster compared to the monomer binding, whereas the apparent dissociation rate became slower with increasing protofibrils size, 2.6 ± 1.0 × 10^−3^ s^−1^ for the small protofibrils and 9.4 ± 2.7 × 10^−4^ s^−1^ for the large.

The apparent affinity of lecanemab for the Aβ fibrils was determined to be 1.8 ± 0.93 nM, approximately twofold and 11-fold lower than for small and large protofibrils, respectively. The weaker apparent affinity, compared to the binding to the protofibrils, was mainly due to an approximately 3.5–5 times slower apparent association rate, indicating that lecanemab prefer protofibrils over fibrils. Aducanumab had approximately 10- to 20-fold slower apparent association and 5- to 10-fold slower apparent dissociation rates when binding to fibrils compared to the protofibril binding, but the apparent affinities for the two Aβ species were similar. Gantenerumab, unlike both lecanemab and aducanumab, had the highest apparent affinity for fibrils, compared to monomer and protofibrils, with a K_D1_ = 0.69 ± 0.16 nM. The higher affinity was driven by a slower apparent dissociation rate, which was approximately two orders of magnitude slower than measured for gantenerumab’s binding to small protofibrils and one order of magnitude slower than the binding to large protofibrils.

## Discussion

In this paper, the binding properties of the anti-Aβ antibodies lecanemab, aducanumab, and gantenerumab were investigated. Since aducanumab and gantenerumab were produced from publicly accessible sequence information, subtle differences of these analogues to the original antibodies could exist. Pure fractions of different Aβ species were prepared and antibody binding was characterized utilizing three different assays: inhibition ELISA and SPR on synthetic Aβ and immunodepletion on synthetic Aβ and AD brain-derived Aβ. The definitions of aggregated species of Aβ in the field are diverse, as well as the methods used for analysis, and therefore direct comparisons between studies are not feasible. Herein, we have analyzed the binding properties of the different antibodies to different Aβ species such as monomers, oligomers, small and large protofibrils, and fibrils. To our knowledge, this is the first time the binding profiles of these antibodies have been compared side-by-side against different species of Aβ.

These antibodies have previously been described as high affinity binders to aggregated Aβ promoting Aβ removal by Fcγ receptor-mediated phagocytosis, while showing lower affinity to monomers [21, 25, 33, 40]. As described herein, the antibodies differ in their selectivity to different soluble Aβ species versus insoluble Aβ fibrils. These differences may have implications on both clinical efficacy and safety readouts reported on these antibodies [21–24].

Aducanumab had the weakest binding to monomers of all antibodies examined, with a *K*_D_ of 7.3 µM as demonstrated by SPR. Although weaker than previously reported *K*_D_ values of 23 and 17 nM [22, 25], gantenerumab was a comparatively stronger monomer binder, with a *K*_D_ of 1.3 µM. This was also seen by inhibition ELISA, where gantenerumab had an IC_50_ of 2.6 µM compared to IC_50_ values above 25 µM for both lecanemab and aducanumab. Compared to lecanemab, which showed a high selectivity for protofibrils versus monomers with a ~ 2300- and ~ 14,300-fold stronger binding to protofibrils (small and large, respectively) than to monomers when analyzed by SPR, gantenerumab showed a ~ 200- and ~ 500-fold selectivity for small and large protofibrils over monomers. The discrepancies regarding binding properties to monomers of gantenerumab compared to previously published data may be due to differences in methodology as well as the quality of the monomer source. Presence of small contaminating amounts of aggregated species in a monomer preparation may have a significant impact on the results. Overall, our data was consistent with previous published results demonstrating that aducanumab has lower affinity for monomeric Aβ than gantenerumab [22].

Lecanemab and gantenerumab demonstrated similar binding strength to 6- to 12-mer oligomers with increased affinity with increasing oligomer size. Gantenerumab bound 2- to 3-mer oligomers stronger than both lecanemab and aducanumab. Aducanumab showed weak binding to soluble oligomers of all sizes. These data are similar to findings by Arndt et al. [22]. They studied the impact of valency on affinity using multi-antigen peptides (MAPs) of branched peptides with different number of copies of Aβ1-15. Aducanumab, unlike gantenerumab, required a larger number of copies of Aβ for efficient binding which is consistent with aducanumab’s dependence of avidity. In this study, gantenerumab bound dimeric and tetrameric MAPs with sub-nanomolar affinities.

Lecanemab showed stronger binding to both small and large soluble protofibrils compared to aducanumab and gantenerumab. Inhibition ELISA confirmed SPR data, although the differences were smaller, which potentially could be explained by limitations in the sensitivity of the ELISA method. Immunodepletion of in vitro generated Aβ protofibrils supported the results from inhibition ELISA. Lecanemab was the antibody that most efficiently immunodepleted synthetic protofibrils, as compared to the other two antibodies. Approximately 50- to 100-fold higher concentration of aducanumab and a tenfold higher concentration of gantenerumab were required to achieve the same protofibril depletion efficiency as with lecanemab. Immunodepletion of protofibrils from soluble AD brain extracts demonstrated that lecanemab and gantenerumab were equally efficient, while aducanumab was less efficient.

The divergence could potentially be explained by the use of synthetic Aβ species as surrogate for Aβ target in human AD brain. The human brain is most likely composed of complex mixtures of different species of Aβ including aggregates of various sizes, N- and C-terminal truncations, and posttranslational modifications. In addition, studying antibody binding to isolated Aβ species may not reflect the dynamics that all species are represented at the same time. It will be important to further investigate the binding profiles of these antibodies across a wide spectrum of soluble versus insoluble Aβ species isolated from human AD brain and their capacity to neutralize Aβ-mediated toxicity.

SPR analysis of aducanumab also revealed different binding kinetics to Aβ. The binding was driven by a very fast association rate rather than a slow dissociation rate. The association rate of aducanumab, when binding to aggregated forms of Aβ, was close to 100-fold faster than that of lecanemab and gantenerumab. For gantenerumab, the dissociation rate was progressively slower as the Aβ aggregates became larger whereas the association rate was unchanged. For all three antibodies, the association rate was faster to protofibrils compared to fibrils, but lecanemab was distinctive in that the dissociation rate was slow and unaffected by the size of the Aβ aggregates. When comparing the dissociation rate constants, it was apparent that aducanumab had a substantially slower dissociation rate when bound to fibrils than when bound to protofibrils. The slower dissociation rate indicated that aducanumab would remain bound longer to fibrils than to protofibrils due to the faster dissociation rate for protofibril binding. Likewise, gantenerumab had a strong preference for the fibrils versus protofibrils. The relative selectivity of gantenerumab for fibrils over small protofibrils was approximately tenfold. In line with Bohrmann et al. [25], our data confirms that gantenerumab had a binding preference to fibrils compared to soluble oligomers.

Moreover, both aducanumab and gantenerumab have been reported to show strong immunostaining within the dense core of amyloid plaques [21, 25] further supporting these antibodies as strong plaque-binders. A clear difference was that lecanemab’s binding to protofibrils was approximately tenfold stronger than its binding to fibrils. These data are supported by our previous in vitro binding analysis revealing that mAb158, the murine precursor of lecanemab, bound approximately 10–15-fold better to Aβ protofibrils compared to Aβ fibrils [18]. Nevertheless, the binding to Aβ fibrils is strong enough to mediate plaque clearance as demonstrated in the phase 2b clinical trial.

In a publication by Linse et al. [41], the impact of aducanumab, bapineuzumab, solanezumab, and gantenerumab on Aβ aggregation kinetics was studied. The authors found, based on this modeling, that aducanumab selectively reduced the secondary nucleation rate, solanezumab selectively inhibited primary nucleation, and bapineuzumab and gantenerumab acted by reducing elongation of fibrils. The effect by aducanumab was caused by the antibody’s interaction with Aβ species involved in secondary nucleation along the surface of fibrils, leading to reduction of oligomers. This could explain the clinical efficacy of aducanumab versus the other three antibodies. However, these observations should be interpreted with some caution, but the findings by Linse et al. provide interesting hypotheses for how anti-Aβ antibodies might act.

One of the most common adverse events following treatment of patients with Aβ antibodies is the development of ARIA-E. In addition to antibody dose and APOE genotype, the selectivity and affinity for soluble or fibrillar Aβ and antibody isotype appear to be of importance for the risk of developing ARIA-E. For instance, crenezumab (IgG4) has triggered less ARIA-E compared to most antibodies of the IgG1 subclass and antibodies with preferential binding to fibrillar over soluble Aβ species are potentially more likely to engage fibrillar Aβ deposited in CAA. Such binding to CAA may increase the risk for ARIA-E, but this warrants further investigation.

In conclusion, using different in vitro binding assays, lecanemab showed the most pronounced preference for soluble Aβ protofibrils versus monomeric and fibrillar forms of Aβ in comparison to the other two Aβ antibodies investigated in this study. Lecanemab’s preferential and strong binding to Aβ protofibrils may explain the difference in clinical efficacy and lower ARIA-E frequency as compared to aducanumab and gantenerumab.

## Supplementary Information

Below is the link to the electronic supplementary material.

Supplementary file1 (TIF 122 KB)

Supplementary file2 (DOCX 51 KB)

Supplementary file3 (PDF 2359 KB)

Supplementary file4 (PDF 509 KB)

Supplementary file5 (PDF 509 KB)

Supplementary file6 (PDF 467 KB)

Supplementary file7 (PDF 509 KB)

Supplementary file8 (PDF 509 KB)

Supplementary file9 (PDF 469 KB)

Supplementary file10 (PDF 440 KB)

Supplementary file11 (TIF 139 KB)
