# Supplementary figures and images for: Lecanemab, Aducanumab, and Gantenerumab — Binding Profiles to Different Forms of Amyloid-Beta Might Explain Efficacy and Side Effects in Clinical Trials for Alzheimer’s Disease
Source: Neurotherapeutics. 2022 Oct 17;20(1):195–206. doi: 10.1007/s13311-022-01308-6 (PMC10119362; doi:10.1007/s13311-022-01308-6)

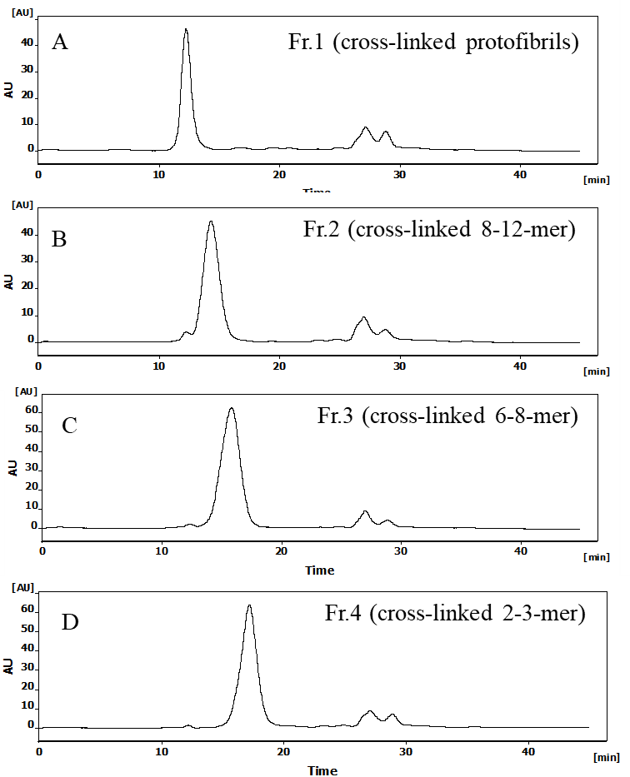

Supplement: Supplementary file 1 — Supplementary file1 (TIF 122 KB) [file 13311_2022_1308_MOESM1_ESM.tif]

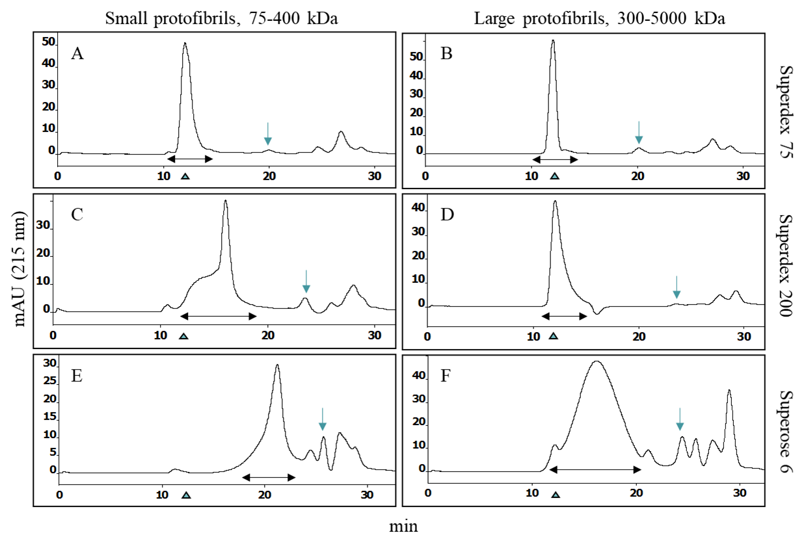

Supplement: Supplementary file 11 — Supplementary file11 (TIF 139 KB) [file 13311_2022_1308_MOESM11_ESM.tif]
