# Supplementary material for: Lecanemab, Aducanumab, and Gantenerumab — Binding Profiles to Different Forms of Amyloid-Beta Might Explain Efficacy and Side Effects in Clinical Trials for Alzheimer’s Disease
Source: Neurotherapeutics. 2022 Oct 17;20(1):195–206. doi: 10.1007/s13311-022-01308-6 (PMC10119362; doi:10.1007/s13311-022-01308-6)
Supplement: Supplementary file 2 — Supplementary file2 (DOCX 51 KB) [file 13311_2022_1308_MOESM2_ESM.docx]

**Supporting information**

The definition of Aβ protofibrils used herein is aggregated Aβ1-42 that remains in the supernatant after centrifugation at 16 000 x g and that by size-exclusion chromatography (SEC) elutes in the void volume of a Superdex 75 column and has a molecular weight larger than >75 kDa. The Aβ protofibril batches were characterized for species heterogeneity using analytical SEC. Two batches of protofibrils were identified as small and large protofibrils respectively, based on their different SEC profiles. Columns with different size ranges demonstrated distinct elution profiles of small and large protofibrils that were not overlapping in size. Both these protofibril batches eluted in the void volume on a Superdex 75 column and neither of them contained monomers, which would elute at 20 min if present (Figure S1A and B). On a Superdex 200 column, the small protofibril batch eluted over a broad time range with a major distinct peak at a retention time of 18 min that corresponded to an estimated size of approximately ~200 kDa according to a globular protein size standard calibration curve (Figure S1C). The large protofibrils eluted in the void of this column, verifying this batch to be larger than >600 kDa (Figure S1D). The sizes were further validated with a Superose 6 column (Figure S1E and F). On this column the large protofibril batch displayed as a heterogenous population consisting of large soluble species ranging from 300 kDa (eluting close to the void) to approximately 5000 kDa.

The oligomers were prepared by short incubation of Aβ1-42 monomers followed by cross-linking using photo-induced cross-linking (PICUP) as described in material and methods. The mixture of cross-linked Aβ species were separated on a Superdex 75 increase 3.2/300 column, and four fractions were collected (Figure S2). The molecular sizes were determined using a calibration curve with molecular standard proteins. Oligomeric species of Aβ may separate with a different retention time compared to globular proteins therefore sizes are only estimates. Fraction 1 eluted in the void and correspond to cross-linked Aβ protofibrils, fraction 2 eluted as octamer to dodecamer (8-12-mer), fraction 3 eluted as hexamer to octamer (6-8-mer) and fraction 4 eluted as dimer to trimer (2-3-mer). The concentration was determined against a protofibril standard curve with known concentration using SEC.

**Figure legends**

**Fig S1** SEC profiles of small (A, C, E) and large (B, D, F) Aβ protofibrils analyzed on three different SEC columns, Superdex 75 increase 3.2/300 (A and B), Superdex 200 increase 3.2/300 (C and D) and Superose 6 increase 3.2/300 (E and F). Absorbance was measured at 215 nm and retention times for protofibrils indicated as horizontal arrows and monomers as vertical arrows.

**Fig S2** Chromatograms from SEC purified cross-linked Aβ oligomers separated on a Superdex 75 increase 3.2/300 column. Fr. 1: cross-linked protofibrils (A), Fr. 2: cross-linked 8-12-mer (B), Fr. 3: cross-linked 6-8-mer (C), Fr. 4: cross-linked 2-3-mer (D). The absorbance was measured at 215 nm. Peaks eluting at a retention time >25 min are buffer peaks.
